# A comparative study between staple line reinforcement during laparoscopic sleeve gastrectomy and no reinforcement: an Egyptian experience

**DOI:** 10.1007/s00464-023-10497-w

**Published:** 2023-10-27

**Authors:** Mohamed Abdul Moneim Amin El Masry, Mohamed Sabry Attia

**Affiliations:** 1 Faculty of Medicine, Cairo University, Cairo, Egypt; 2 Faculty of Medicine, Cairo University, Cairo, Egypt

**Keywords:** Obesity, Laparoscopic sleeve gastrectomy (LSG), Early postoperative morbidity, Staple line reinforcement (SLR), Oversewing

## Abstract

**Background:**

Laparoscopic sleeve gastrectomy (LSG) has become an increasingly popular bariatric procedure. LSG still conveys some risks, including early staple line complications such as bleeding and leaks. It has been proposed that staple line complications can be reduced by staple line reinforcement (SLR). This study aimed to compare the short-term efficacy and safety of the SLR during LSG by oversewing versus no SLR in an Egyptian cohort over a period of 11 years.

**Patients and methods:**

This is a retrospective study that analyzed data from patients undergoing LSG by the same surgeon over a period of 11 years. The patients’ early postoperative complications were compared according to performing SLR.

**Results:**

The SLR group showed significantly longer surgery time (*p* = 0.021) and a lower rate of postoperative bleeding (*p* = 0.027). All leakage cases occurred in the non-SLR group (0.7% vs. 0.0%) without statistical significance (*p* = 0.212). The two mortality cases occurred in the non-SLR group. The LOS was comparable in the two groups (*p* = 0.289).

**Conclusion:**

This study confirms the short-term benefits of SLR by oversewing during LSG in terms of a lower incidence of 30-day morbidity, particularly bleeding, and lower rates of reoperation, with a clinically questionable longer operation time.

The continuously rising incidence of obesity poses a significant impact on the healthcare system all over the world [1]. Bariatric surgery is the most effective approach for achieving clinically significant and sustained weight loss in patients with obesity [2].

Among several bariatric procedures, laparoscopic sleeve gastrectomy (LSG) is currently the most popular worldwide owing to its technical simplicity, effectiveness, and safety [3]. Compared to the other surgical procedures, LSG is particularly notable for the little derangement of the gastrointestinal tract (GIT) anatomy, maintaining the intestine intact without excluding portions that lead to malabsorption, or anastomosis. Furthermore, it can be converted into other bariatric procedures [4].

Despite the described advantages, LSG still conveys some risks. Early staple line complications, such as bleeding and leaks, may occur, and their incidence may vary from 1 to 6% [5, 6]. Such complications can be devastating and life-threatening. Besides, they entail additional healthcare-related costs. It has been proposed that staple line complications can be reduced by staple line reinforcement (SLR) [7, 8]. Suture oversewing (SR), glue reinforcement (GR), omentopexy (OP)/gastropexy (GP), and buttressing have been described for SLR [7–10].

Some surgeons still have concerns about SLR, either because of uncertainty about its benefits and/or its financial costs. Moreover, it has been argued that oversewing itself could carry additional risks. The potential for leakage and bleeding could increase due to tearing at the suture penetration point, and the running suture may lead to sleeve stricture and tissue ischemia [11, 12].

Given this controversy, this study aimed to compare the short-term efficacy and safety of the SLR during LSG by oversewing versus non-SLR in an Egyptian cohort over a period of 11 years.

## Patients and methods

This study is a retrospective analysis of prospectively collected data on patients who were consecutively scheduled for LSG by the first author over a period of 11 years. The study was approved by the Research Ethics Committee and performed as per the Declaration of Helsinki.

The patients were recruited for bariatric procedures at the institutions of the study after checking their fitness for the surgery based on the criteria employed by the 1991 NIH consensus [13] and established by the international bariatric surgery societies: the International Federation for the Surgery of Obesity (IFSO), the American Society for Metabolic and Bariatric Surgery (ASMBS), and the European Association for the Study of Obesity (EASO) [14–16]. Patients who underwent LSG based on their choice after dedicated discussion with the surgeon were eligible for the study. All patients were subjected to the routine preoperative clinical examination, which included full history taking, multidisciplinary clinical assessment, laboratory investigations, abdominal ultrasound, and upper GIT endoscopy. Patients with large hiatus hernias or severe gastroesophageal reflux disease (GERD), based on clinical presentation and/or endoscopic assessment, were not candidates for LSG. Written informed consent was obtained from the included patients before surgery. Patients whose medical files included incomplete relevant data were excluded from the study.

The surgery was performed as previously described [17]. In summary, after adequate preoperative preparation of the patients, general anaesthesia was induced. The surgery was performed while the patient was in the reverse Trendelenburg position. The standardized five-trocar technique was followed, and pneumoperitoneum was established. A full fundus mobilization was ensured until the identification of the left crus. After then, the sleeve was created over a 36-Fr bougie, starting about 3–4 cm proximal to the pylorus and proceeding to the His angle.

Drain insertion was routinely employed until the end of 2015. As of 2016, drains were inserted only in selected cases (hypertension, bleeding tendency, adhesions).

SLR was not performed before 2018. The staple line was tested for bleeding by inducing elevated systolic blood pressure coincident with the reduction of pneumoperitoneum pressure to 10 mm/Hg to detect potential bleeding sites. The isolated bleeding points were then clipped. Lastly, a methylene blue leak test was carried out to assess any potential leakage.

As of 2018, we adopted the routine SLR by oversewing. Suture reinforcement was performed using polyglactin 910 3/0 sutures starting at the upper end of the staple line in a through-and-through continuous manner with invagination of the last 5–6 cm of the staple line. This was employed to ensure robust reinforcement of the staple line, minimizing the risk of potential leaks.

Patients were monitored after the operation and motivated for early mobilization. They received the post-operative instructions with the diet and supplementation regimen and follow-up schedules and were informed to seek medical advice in case of any adverse event.

The patients’ demographic, clinical, operative, and postoperative data were recorded and analyzed. The study outcomes were the potential impact of SLR on LSG-associated 30-day bleeding and leakage.

### Statistical analysis

The patients’ data was analysed using the SPSS statistical software (IBM Corp., Armonk, NY, USA), version 28. Numerical data were compared using an independent *t*-test or Mann–Whitney test, as appropriate. Categorical values were compared using the chi-square test. A binary logistic analysis was performed to identify the potential predictors of early postoperative morbidity. A *p*-value less than 0.05 was considered statistically significant.

## Results

The present study included 892 patients who were consecutively recruited for LSG during the period from July 2011 to August 2022 by the same surgeon (the first author). The mean age of the included patients was 35.98 ± 10.25 years, with a higher female prevalence (*n* = 641, 71.9%). The mean preoperative weight was 131.39 ± 25.26 kg, and the mean preoperative BMI was 47.43 ± 7.57 kg/m^2^. The obesity-associated comorbidities were mainly dyslipidemia, hypertension, and type 2 diabetes mellitus (Table 1).

**Table 1:** Baseline demographic data and operative events of the study patients

	Study patients (*n* = 892)	
	Mean ± SD	Range
Age (year)	35.98 ± 10.25	18–60
Baseline weight (kg)	131.39 ± 25.26	80–270
Baseline BMI (kg/m^2^)	47.43 ± 7.57	35.7–102
Total surgery time (min)	66.08 ± 21.52	50–110
Hospital stay (days)	1.094 ± 1.15	1–28

	Count	%
Sex		
Male	251	28.1%
Female	641	71.9%
Comorbidities		
Type 2 diabetes mellitus	90	10.1%
Hypertension	158	17.7%
Dyslipidemia	302	33.86%
Drain insertion		
Yes	402	45.1%
No	409	54.9%
Early postoperative adverse events		
Yes	16	1.79%
No	876	98.21%
Required blood transfusion		
Yes	12	1.35%
No	880	98.65%
Required ICU admission		
Yes	2	0.22%
No	890	99.78%
Re-operation rate		
Yes	8	0.9%
No	884	99.1%
Mortality rate		
Yes	2	0.22%
No	890	99.78%

During the operation, a little less than half of the patients required drain insertion (*n* = 402, 45.1%). The mean operative time was 66.08 ± 21.52 min. The total hospital stay ranged from 1 to 28 days, with a mean of 1.094 ± 1.15 days. Early postoperative adverse events were encountered in 16 patients (1.79%). Six patients had intra-abdominal bleeding, two had intra-abdominal leakage, of whom one was complicated by an intra-abdominal abscess and the other had acute sepsis, and three had intraoperative bleeding and leakage. Other complications were massive pulmonary embolism in one patient and wound bleeding/hematoma in four patients.

Overall, twelve patients (1.35%) required blood transfusion (eight bleeding cases, one case of acute sepsis on top of leakage, and three cases of wound hematoma), two patients (0.22%) required ICU admission (the pulmonary embolism and the acute sepsis cases), and eight patients (0.9%) underwent re-operation (the bleeding and/or leakage cases apart from the three bleeding cases were managed conservatively). The mortality rate was 0.22% (*n* = 2). Table 2 presents the demographic and clinical details of the sixteen cases of early postoperative morbidity.

**Table 2:** Demographic data and early postoperative events of the patients with early postoperative morbidity

	Age (year)	Sex	Comorbidity	Baseline BMI (kg/m^2^)	Surgery Year	Surgery time (min	Adverse events	management	LOS (days**)**	Mortality
Case 1	37	Female	None	43.2	2011	63	Intra-abdominal bleeding	Open exploration	3	No
Case 2	38	Female	None	44.4	2012	70	Intra-abdominal bleeding and Leakage	Open exploration	28	No
Case 3	25	Male	None	44.2	2013	71	Intra-abdominal bleeding	Laparoscopic exploration	3	No
Case 4	31	Female	None	47.1	2013	81	Intra-abdominal leakage with abscess formation	Laparoscopic exploration	4	No
Case 5	48	Female	Dyslipidemia	61.9	2013	62	Wound hematoma	Conservative	2	No
Case 6	32	Male	Hypertension, dyslipidemia	63.8	2014	50	Intra-abdominal bleeding	Laparoscopic exploration	3	No
Case 7	50.1	Male	None	50.4	2014	64	Intra-abdominal bleeding	Conservative	2	No
Case 8	39	Male	Hypertension, dyslipidemia	57.7	2014	62	Wound hematoma	Conservative	2	No
Case 9	30	Male	Hypertension, dyslipidemia	43.1	2015	37	Massive pulmonary embolism	Anticoagulation Therapy, Thrombolytic Therapy	5	Yes
Case 10	34	Female	Hypertension, T2DM, dyslipidemia	51.1	2016	65	Intra-abdominal bleeding	Conservative	2	No
Case 11	45	Female	None	46.4	2017	52	Intra-abdominal bleeding	Conservative	2	No
Case 12	50	Female	Hypertension, dyslipidemia	63.3	2017	53	Intra-abdominal leakage	Open exploration	11	Yes
Case 13	29	Female	None	50.6	2018	52	Intra-abdominal bleeding and Leakage	Laparoscopic exploration	14	No
Case 14	34	Female	Dyslipidemia	63.7	2018	50	Intra-abdominal bleeding and Leakage	Laparoscopic exploration	13	No
Case 15	36	Female	None	43	2019	71	Wound subcutaneous bleeding	Conservative	3	No
Case 16	34	Female	Hypertension, T2DM, dyslipidemia	44.4	2020	110	Wound subcutaneous bleeding	Conservative	3	No

At the 6-month follow-up, the mean patients’ BMI was 34.61 ± 6.96 kg/m^2^, and the mean EBWL% was 63.8 ± 15.55%.

Since October 2018, we have implemented routine oversewing for all cases (211 patients; 23.72%). A comparison of patients who had SLR to those who did not is shown in Table 3. Patients in both groups had comparable baseline characteristics. There was a significantly longer surgery time (*p* = 0.021) and significantly lower rate of drain insertion (*p* < 0.001) in the SLR group. The SLR group showed a lower rate of overall early postoperative morbidity (0.9% vs. 2.15). However, without statistical significance (*p* = 0.289). Concerning bleeding, it was obvious that the rate considerably decreased after applying SLR (0.0% vs. 1.3). The difference reached the level of significance when assessing the likelihood ratios (*p* = 0.027). All leakage cases occurred in the non-SLR group (0.7% vs. 0.0%) without statistical significance (*p* = 0.212). Speaking of bleeding and leakage together, statistically lower rates were shown in the SLR group (0.0% vs. 1.6%, *p* = 0.014). The two mortality cases occurred in the non-SLR group. The LOS was comparable in the two groups (*p* = 0.289).

**Table 3:** Baseline demographic data and operative events of the study patients

	Study patients (*n* = 892)		
	Non-SLR group *N* = 681	SLR group *N* = 211	*p*-value
	Mean ± SD	Mean ± SD	
Age (year)	35.83 ± 9.6	36.47 ± 12.1	0.486^a^
Baseline weight (kg)	128.13 ± 22.72	126.31 ± 25.36	0.323^a^
Baseline BMI (kg/m^2^)	48.51 ± 8.51	47.01 ± 7.91	0.354^a^
Total surgery time (min)	65.02 ± 19.99	69.48 ± 25.61	0.021*^a^
6-month BMI (kg/m^2^)	35.09 ± 7.15	34.2 ± 5.03	0.321^a^
Hospital stay (days)	1 (1–28)	1 (1–3)	0.289^b^
	Count (%)	Count (%)	
Sex			
Male	197 (28.9)	54 (25.6)	0.346^c^
Female	484 (71.1)	157 (74.4)	
Comorbidities			
Type 2 diabetes mellitus	64 (9.39)	26 (12.32)	0.218^c^
Hypertension	126 (18.51)	32 (15.17)	0.267^c^
Dyslipidemia	232 (34.07)	70 (33.18)	0.293^c^
Drain insertion (Reviewer # 1, Comment # 4)			
Yes	375 (55.1)	27 (12.8)	< 0.001^b^*
No	306 (44.9)	184 (87.2)	
Early postoperative adverse events			
Yes	14 (2.1)	2 (0.9)	0.289^b^
No	667 (97.9)	209 (99.1)	
Bleeding			
Yes	9 (1.3)	0 (0)	0.093^c^, 0.027 ^d^*
No	672 (98.7)	211 (100)	
Leakage			
Yes	5 (0.7)	0 (0)	0.212 ^c^
No	676 (99.3)	211 (100)	
Required blood transfusion			
Yes	11 (1.6)	1 (0.5)	0.291^c^
No	679 (98.4)	210 (99.5)	
Required ICU admission			
Yes	2 (0.3)	0 (0)	0.431^c^
No	679 (99.7)	211 (100)	
Re-operation rate			
Yes	8 (1.2)	0 (0)	0.114^c^, 0.037 ^d^*
No	673 (98.8)	211 (100)	
Mortality rate			
Yes	2 (0.3)	0 (0)	0.431^c^
No	679 (99.7)	211 (100)	

*Statistically significant

^a^Independent *t*-test

^b^Mann–Whitney test

^c^Chi-square test

^d^Chi-square likelihood test

All the patients’ baseline clinical and operative characteristics were tested for the potential association with the occurrence of early postoperative morbidity. Binary logistic regression analysis demonstrated that only the patient’s BMI (OR = 1.05; CI 1–1.102; *p* = 0.048) and the presence of hypertension (OR = 0.256; CI 0.084–0.782; *p* = 0.017) significantly predicted the rate of postoperative morbidity in the non-SLR group but not in the SLR group (*p* = 0.657 and 0.619, respectively). It is worth noting that drain insertion did not show a significant association with early postoperative morbidity in either group (*p* = 0.486 and 0.998, respectively).

## Discussion

Laparoscopic sleeve gastrectomy has been reported for its safety, and it has been a growingly satisfying obesity management procedure for patients and surgeons [18–21]. However, perioperative risks remain. In addition to the risks attributed to the long staple line [22], patients with obesity are already a vulnerable group with an elevated risk of perioperative morbidity [23]. This is a retrospective analysis of an Egyptian cohort who underwent LSG by a single surgeon over a period of 11 years. The rates of 30-day postoperative complications and mortality were 1.79% and 0.22%, respectively. These rates are within the previously reported ranges of early morbidity (1.2 to 7.9%) and mortality (0.0–0.3%) [18, 20, 21].

There remain aspects for better outcomes after bariatric surgery. In an effort to prevent staple line-related complications, staple line reinforcement has been advocated. However, it is still an area of much debate concerning its safety and cost–benefit ratio [24–31].

SLR is performed by several methods, but whatever the technique used, SLR certainly increases the operation time [10]. This was shown in our study, where the operation time was significantly prolonged in the SLR group. However, the clinical significance of the time difference is questionable since we had a mean difference of less than 5 min. Previous studies reported variable differences in surgery time. In the study by Albanopoulos and colleagues [32], there was no significant difference between the two groups. Other studies reported a mean difference of up to 15.5 [29] and 16.65 [24] minutes. In fact, the operation time is influenced by several factors, such as the technique of the surgery, including whether SLR is performed or not and the type of SLR used, the skillfulness and experience of the surgeon, and the collaboration of the surgery team. This could explain the described variation among different studies.

Leakage and bleeding are still the most ominous early post LSG complications [33]. Being life-threatening, many surgeons perform LSG with SLR in an attempt to reduce postoperative leakage and bleeding. This was supported by the International Sleeve Gastrectomy Expert Panel consensus statement that recommends the routine use of SLR during LSG based on the unanimous agreement of the panel members who experienced LSG on more than 12,000 patients [34]. However, some surgeons still argue against SLR and raise questions regarding its costs, benefits [35–37], and safety [11, 12].

In this work, there were obviously lower rates of bleeding and leakage in the SLR (1.3% and 0.7%, respectively, vs. 0.0% for both). The present study, however, did not show statistical significance in the difference in leakage rates. This is likely attributable to the small number of patients with leakage. In line with our results, the efficacy of SLR in reducing bleeding was confirmed by several studies that concluded the benefit of SLR in this respect, whatever the method of reinforcement [25, 30, 35, 38–41]. Close to our findings, Kwiatkowski et al. [25], reported bleeding and leakage incidences of 4.6 and 2.3%, respectively, in the SLR group compared to 0% in the SLR group. Likewise, ElGohary et al. [40] declared incidences of 4% and 2% versus 1.5% and 0% in the two groups, respectively. Also, D’Ugo et al. found a bleeding rate of 13.7% in the non-SLR group compared to 1.4% in the SLR group [41]. The better outcome in the present work is likely related to the larger sample size compared to the described studies [25, 40] or the method of SLR [41]. In this study, oversewing was used to reinforce the staple line. Varban et al. [42] found that oversewing was the only SLR technique that decreased the bleeding incidence. The same conclusion was reached by Diab et al. [24], Aiolfi et al. [29], Casella et al. [43], and Sroka et al. [44]. Aggarwal et al. [45] reported that oversewing via invaginating sutures totally eliminated bleeding. Variations among different studies could also be attributed to the learning curve, used instruments, postoperative care, and patient comorbidities.

In the present work, the lower rate of early postoperative morbidity reflected a significantly lower reoperation rate. This is consistent with the results of Hany et al. [22], Diab et al. [24], ElGohary et al. [40] and Dapri et al. [46]. This study showed that SLR did not influence the LOS. This is consistent with the study of Wu et al. who reported comparable LOS in the two groups [47].

It should be highlighted that despite the significantly lower rates of drain insertion in the SLR group, this factor may not have a direct impact on the occurrence of postoperative morbidity in either group, as shown from the regression analysis.

Acknowledging the potential challenges associated with drains and their non-alignment with Enhanced Recovery After Surgery (ERAS) protocols, we found that, within the characteristics of our patient cohort, the anticipated negative consequences of drain usage may not have manifested prominently enough to significantly influence the observed patient outcomes. This might reflect the effective management and monitoring of drain-related factors in our study, contributing to the lack of a statistically significant association with postoperative morbidity.

The present work's findings emphasize the advantages of routine oversewing during SLR. In the context of high costs, oversewing provides the benefits of SLR with relatively lower costs than other SLR methods such as buttressing and glueing. Thus, it is suitable for developing countries like Egypt. This study did not estimate the precise cost differences between the two groups. Even though the added cost of oversewing is likely non-comparable to the evident oversewing-associated benefits in terms of lower rates of morbidity and reoperation, it could lead to even more costs.

The present work is limited by its retrospective design. Also, some of the patients who had early postoperative complications in the non-SLR group underwent surgery during the initial learning curve of the surgeon, while all patients in the SLR group had the surgery performed after at least 7 years of experience. However, this is one of the few studies to include a large cohort of patients undergoing LSG in Egypt, and the study sheds light on a still debatable area. Further head-to-head prospective comparative studies are warranted.

## Conclusion

This study confirms the short-term benefits of SLR by oversewing during LSG in terms of a lower incidence of 30-day morbidity, particularly bleeding, and lower rates of reoperation, with a clinically questionable longer operation time.
